# Vitamin D status and vitamin D deficiency risk factors among pregnancy of Shanghai in China

**DOI:** 10.1186/s12884-021-03889-0

**Published:** 2021-06-18

**Authors:** Chun Yang, Wu Jing, Sheng Ge, Wenguang Sun

**Affiliations:** 1 Department of Nutrition and Food Hygiene, School of Public Health, Capital Medical University, Beijing, China; 2 Beijing Key Laboratory of Clinical Epidemiology, Beijing, China; 3 Beijing Key Laboratory of Environmental Toxicology, Beijing, China; 4 Clinical Nutrition Department, The First Affiliated Hospital of Zhejiang Chinese Medical University, Zhejiang Province, China; 5 Clinical Nutrition Department, Shanghai Sixth People’s Hospital, Affiliated to Shanghai Jiao Tong University, Shanghai, China; 6 School of Medicine, The International Peace Maternity and Child Health Hospital, Shanghai Jiao Tong University, Shanghai, China; 7 Shanghai Key Laboratory of Embryo Original Diseases, Shanghai, China; 8 Shanghai Municipal Key Clinical Specialty, Shanghai, China

**Keywords:** Vitamin D status, Pregnancy, Risk factors, Shanghai, China

## Abstract

**Background:**

There is increasing awareness that vitamin D deficiency in pregnant women may be associated with several adverse effects for the mother and newborn. The risks for vitamin D deficiency are unclear. This study was to assess vitamin D nutritional status and vitamin D deficiency risk factors among pregnant women in Shanghai in China.

**Methods:**

This study is a cross-sectional study conducted in the Sixth Affiliated People’s Hospital of Shanghai Jiao Tong University. A total of 953 healthy pregnant women participated, serological examinations and other variables included serum 25-hydroxyvitamin D [25(OH)D], total blood cholesterol (TCh), high-density lipoprotein (HDL), low-density lipoprotein (LDL), and very-low-density lipoprotein (VLDL) cholesterol, triglycerides at the first antenatal visit (12–14 weeks) pregnancy parity and age, body mass index (BMI) before pregnancy, and completed OGTTs test. Associations between vitamin D deficiency and possible predictors (age group, pre-pregnancy BMI, parity, and gestational hyperlipemia) were assessed with a multinomial logistic regression analysis. And also used to investigate the effects of 25(OH)D and the other variables on the occurrence of gestational diabetes mellitus.

**Results:**

The mean vitamin D level of pregnancy was 16 (a range from 11 to 21) ng/ml, and severe vitamin D deficiency was 31.8% (303); vitamin D deficiency was 40.7% (388); vitamin D insufficiency was 25.1% (239); normal vitamin D was 2.4%(23). Vitamin D deficiency risk factors were age over 30, parity over 2, overweight, obese, and hyperlipemia. The increasing level of vitamin D nutritional status in pregnancy is significantly related to reducing gestational diabetes mellitus. Vitamin D deficiency is a risk factor for gestational diabetes mellitus.

**Conclusions:**

It is a high prevalence of vitamin D deficiency in Chinese pregnancy in Shanghai. Aging more than 30 years, the parity of more than 2, overweight and obesity, and hyperlipemia are risk factors for vitamin D deficiency. Vitamin D deficiency is a risk factor for gestational diabetes mellitus. Public health strategies to prevent vitamin D deficiency should focus on those risks to promote health pregnancy of Shanghai in China.

## Background

Vitamin D is an essential fat-soluble vitamin that acquired through dietary intake and cutaneous synthesis under ultraviolet radiation [1]; the primary vitamin D function promotes calcium absorption in the gut and maintains adequate serum calcium and phosphate concentrations to enable normal bone mineralization [2, 3], and also has other roles in the body, including reduction of inflammation as well as modulation of such processes as cell growth, neuromuscular and immune function, and glucose metabolism [4]. Many studies have reported inverse associations between vitamin D status, such as depression [5], breast cancer, type 2 diabetes, type 1 diabetes [6], cardiovascular diseases [7], autoimmune diseases [8], infections [9], and autism [10]. Vitamin D deficiency is a public health issue in developing and developed nations. Pregnant women have been considered high-risk groups whose prevalence of vitamin D deficiency ranges from 20 to 90% [11, 12]. Moreover, vitamin D insufficiency in pregnant women may be associated with several adverse effects for the mother and newborn [13], vitamin D insufficiency among mothers may lead to bone impairment and osteoporosis [14], low vitamin D levels are associated with depressive symptoms during pregnancy, and postpartum depression [15] also related to an increased risk of gestational diabetes, preeclampsia, small for gestational age infants, and low birth weight infants [16].

Vitamin D status and metabolism during pregnancy affect calcium absorption from the intestinal tract and outdoor activities. Previous studies suggested that risk factors for vitamin D deficiency during pregnancy, including vegetarians, women with limited sun exposure, those who live in cold climates, reside in northern latitudes, or wear sun and winter protective clothing, and ethnic minorities, especially those with darker skin [17–19]. The Chinese traditional dietary pattern is mainly based on vegetables and grain, which raised low vitamin D levels. Shanghai is one of the largest cities in China with more than 20 million people, and it is the location of East China at 31 degrees north latitude. Recent evidence suggests that pregnant women in Shanghai were generally deficient in vitamin D status [20, 21], from 90.5% to 98.4% of these women had serum 25(OH)D levels below 30 ng/mL and the causes of high vitamin D deficiency among high-risk groups in this region remain insufficiently known.

The risks of vitamin D deficiency in Shanghai have been done, and data from this population remain poorly understood. Vitamin D supplementation of 10 μg/day during pregnancy, suggested by the Chinese Nutrition Medicine Association, was equal to the adult’s recommended amount. Also, it was reported, although the data is limited, that the vast majority of Chinese women do not, in reality, take vitamin D supplementation during pregnancy [22]. Therefore, a better understanding of vitamin D status and identifying risks of vitamin D deficiency, particularly in regions where pregnancy is at risk of suboptimal vitamin D status, is critical. This study aimed to determine vitamin D status and its determinants among pregnancy in Shanghai in China.

## Methods

The study was carried out following the guidelines proposed in the Declaration of Helsinki. All procedures involving human subjects went through ethical approval by the Ethics committee of Sixth Affiliated People’s Hospital of Shanghai Jiao Tong University (2,016,016). All participants signed an informed consent form. All pregnant women in their late second or early third trimester of pregnancy in the obstetrics department from December 2016 to April 2017 were identified, and their data were reviewed retrospectively. Individuals who had the presence of symptoms or signs of active infectious disease or chronic illness; abnormal renal/liver function (serum glutamate pyruvate transaminase [SGPT] ≥ 18 IU/L, serum glutamate oxaloacetate [SGOT] ≥ 16 IU/L, serum urea concentration ≥ 7.5 mmol/L, serum alkaline phosphatase concentration ≥ 280 IU/L); the presence of Hepatitis B surface antigen (HBsAg) or antibodies to Hepatitis C virus (HCV) or Human Immunodeficiency Virus (HIV) and vitamin D supplement use in the past six months were excluded.

Our study selected serological examinations and other variables included serum 25-hydroxyvitamin D [25(OH)D], total blood cholesterol (TCh), high-density lipoprotein (HDL), low-density lipoprotein (LDL) or very-low-density lipoprotein (VLDL) cholesterol, triglycerides at the first antenatal visit (12–14 weeks) pregnancy parity and age, body mass index (BMI) before pregnancy. Vitamin D status was determined with serum 25-hydroxyvitamin D [25(OH)D] levels [23], serum 25(OH) D levels were measured by electrochemiluminescence immunoassay with Roche Cobas 6000’s module e601 (Roche Diagnostics GmbH, Mannheim, Germany), the functional sensitivity of 25(OH)D was ≤ 3 ng/mL (< 7.5 nmol/L), the coefficient of variation (CV) within batch was 1.7–7.5%, and the CV between batches was 2.2–13.6%. The clinical cutoffs of 25(OH)D as following less than 12 ng/ml, between 12 and less than 20 ng/ml, and between 20 and less than 30 ng/ml were regarded to present severe vitamin D deficiency, vitamin D deficiency, and vitamin D insufficiency, concentrations between 30 and 50 ng/ml were considered normal [24, 25]. TCh, HDL, LDL, VLDL cholesterol, and triglycerides were measured with an automatic biochemical analyzer (Hitachi 7600 120, Hitachi, Japan). Women with < 280 mg/dL (7.28 mmol/L) at term or ≥ 280 mg/dL TCh were considered as maternal physiological hypercholesterolemia (MPH) or maternal supraphysiological hypercholesterolemia (MSPH) [26]. The cutoff point of 280 mg/dL TCh for MSPH was established for the following reasons: (1) all assays performed in samples from women with ≥ 280 mg/dL TCh were found to exhibit significant differences from those from women with < 280 mg/dL TCh, (2) patients with a TCh level approaching this point are associated with fetal fatty streaks, and (3) this value is above the mean TCh level (~ 247 mg/dL, the range is from 184–315 mg/dL) considered normal in pregnancy of different groups [27]. According to the adult BMI classification standards for the Chinese population [28], pre-pregnancy BMI, calculated as weight (kg) divided by height (m) squared, was categorized into four groups: underweight (< 18.5 kg/m^2^), normal (18.5 kg/m^2^ to 24.0 kg/m^2^), overweight (24.0 kg/m^2^ to 28.0 kg/m^2^) and obese (≥ 28.0 kg/m^2^). Screening for Gestational diabetes mellitus (GDM) used 75 g oral glucose tolerance test (OGTT) was ordered at 24–28 weeks’ gestation. It may be repeated in the third trimester for women with normal test results if there is suspicion of GDM. Diagnosis of GDM was made with either fasting plasma glucose levels ≥ 5.1, plasma glucose levels for 1 h after glucose intake ≥ 10.0, or plasma glucose levels for 1 h after glucose intake ≥ 8.5 mmol/L [29].

### Statistical analysis

Participants were classified into sub-groups according to hypothesized vitamin D status predictors: age group, parity, pre-pregnancy BMI, gestational hyperlipemia, and gestational diabetes. To determine associations between vitamin D levels and the hypothesized predictors, a Kruskal–Wallis test followed by a Mann–Whitney U test was conducted as Kolmogorov–Smirnov test did not reveal a normal distribution for most sub-groups. Test for interaction in the logistic regression model was used to compare odd ratios between the analyzed subgroups. Associations between vitamin D deficiency and possible predictors (age group, pre-pregnancy BMI, parity, and gestational hyperlipemia) were assessed with a multinomial logistic regression analysis. Logistic-regression models were used to investigate the effects of 25(OH)D and the other variables on the occurrence of gestational diabetes mellitus. The multivariable regression model containing the other variables, including age, pre-pregnancy BMI, parity, TCh, HDL, LDL, and triglycerides risk associated with gestational diabetes mellitus, is reported according to clinical cutoffs of 25(OH)D. Data were analyzed with the statistical software package SAS version 9.2. The primary analysis is descriptive, and the 95% Cite significance level was set at P < 0.05 using two-sided tests.

## Results

Our study found that the level of vitamin D of Chinese pregnant women in Shanghai was 16 (11–21) ng/ml, and 31.8% (303) pregnant women had severe vitamin D deficiency; 40.7% (388) had vitamin D deficiency; 25.1% (239) had vitamin D insufficiency; only 2.4% (23) had normal vitamin D; None were found to have vitamin D above 50 ng/ml. Our study found that the level of vitamin D increased with age groups (t = 8.5, *p* = 0.04); Vitamin D deficiency decreased with age groups (X^2^ = 17.6, *p* = 0.04). As the parity of Chinese pregnancy in Shanghai increased, the level of vitamin D was decreased (t = 2.7, *p* = 0.04), and Vitamin D deficiency was increased (X^2^ = 45, *p* = 0.001). As pre-BMI levels increased, the level of vitamin D decreased (t = 14, *p* = 0.003); and vitamin D deficiency is increased (X^2^ = 29, *P* = 0.01). The level of vitamin D in pregnancy of gestational diabetes mellitus was lower than non-gestational diabetes mellitus pregnancy (t = 13, *p* = 0.02), and the prevalence of vitamin D deficiency was higher than non-gestational diabetes mellitus pregnancy (X^2^ = 2.4, *P* = 0.01). There was no statistical significance of other variables (Table 1).

**Table 1:** Characteristics of Chinese pregnant women in Shanghai (*n* = 953)

Variable/group	25(OH)D (ng/ml)			*P* value	Percentage with 25(OH)D (ng/ml)					*P* value
	n	Mean	95%CI		n	< 12	12–19	20–29	30–50	
Total	953	16	11–21		953	31.8% (303)	40.7% (388)	25.1% (239)	2.4% (23)	
Age (years)										
20–25	120	16	15–17	8.5	120	31.7% (38)	42.5% (51)	25.8% (31)	0.0% (0)	17.6
25–30	471	16	15–16		471	36.5% (172)	38.4% (181)	22.5% (106)	2.5% (12)	
30–35	274	17	16–18		274	26.3% (72)	41.6% (114)	29.6% (81)	2.6% (7)	
≥ 35	88	17	15–18	0.04	88	23.9% (21)	47.7% (42)	23.9% (21)	4.6% (4)	0.04
Parity										
0	671	16	15–17	2.7	671	30.7% (206)	41.3% (277)	26.1% (175)	1.9% (13)	45
1	248	15	14–16		248	35.1% (87)	40.7% (101)	23.4% (58)	0.8% (2)	
≥ 2	34	14	12–15	0.04	34	29.4% (10)	29.4% (10)	17.7% (6)	23.5% (80)	0.001
Pre-pregnancy BMI (kg/m^2^)										
Underweight	119	17	16–18	14	119	16.0% (19)	52.1% (62)	28.6% (34)	21.1% (4)	29
Normal	623	16	15–17		623	32.1% (200)	38.2% (238)	27.1% (169)	2.6% (16)	
Overweight	162	15	14–16		162	40.7% (66)	40.7% (66)	17.9% (29)	0.6% (1)	
Obese	49	14	13–16	0.003	49	36.7% (18)	44.9% (22)	14.3% (7)	4.1% (2)	0.01
Gestational diabetes mellitus										
Yes	56	16	15–18	13	56	39.3% (22)	32.1% (18)	25.0% (14)	3.6% (2)	2.4
No	897	15	13–16	0.02	897	31.3% (281)	41.3% (370)	25.1% (225)	2.3% (21)	0.01
Gestational Hyperlipemia										
No	931	16	15–17	0.4	931	31.9% (297)	40.7% (379)	25.1% (234)	2.3% (21)	0.6
Yes	22	15	13–16	0.7	22	27.3% (6)	40.9% (9)	22.7% (5)	9.1% (2)	0.9

Our results of the multinomial logistic regression model for screening for vitamin D deficiency risk factors found that aging over 30, parity over 2, overweight and obesity, gestational hyperlipemia of pregnancy were risk factors for vitamin D deficiency. Risks for presenting with vitamin D deficiency in pregnant women aging over 30 were 3.5 times that of women under 30, and risks for women with over parity 2 of pregnancy were 2.8 times that of women below parity 2. Risks of vitamin D deficiency for overweight and obese and hyperlipemia pregnancies were 2.1 and 2.2 and 1.5 times that of normal pregnancy (Table 2).

**Table 2:** Multinomial logistic regression model for vitamin D deficiency among pregnant of Shanghai in China (*n* = 953)

Variable/group	n	OR	95% CI	*P* value
Age (years)				
< 30	591	ref		
≥ 30	362	3.5	2–4	0.02
Parity				
< 2	921	ref		
≥ 2	32	2.8	2–5	0.03
Pre-pregnancy BMI (kg/m^2^)				
Underweight	119	1.1	1–2	0.41
Normal	623	ref		
Overweight	162	2.1	2–5	0.03
Obese	49	2.2	2–5	0.02
Gestational Hyperlipemia				
No	931	ref		
Yes	22	1.5	1–2	0.04

Logistic regression analyses result of the effect of vitamin D on gestational diabetes showed that pregnant women in insufficient vitamin D had a 7.5 (2–21) times increased odds ratio of GDM compared with sufficient vitamin D; pregnant women in severe vitamin D deficiency and vitamin D deficiency had 11 (3–17) and 8.6 (2–19) times increased odds ratio of GDM compared with vitamin D sufficient respectively (Table 3).

**Table 3:** Multinomial logistic regression model for the effects of 25(OH)D on the occurrence of gestational diabetes mellitus among pregnant of Shanghai in China (*n* = 953)

Clinical cutoffs	Incidence n (%)	Non-adjusted OR (95% CI)	*P* value	Adjust model OR (95% CI)	*P* value
< 12(ng/mL)	22 (2.3%)	11.2 (3–18)	0.001	11 (3–17)	0.001
12–19 (ng/mL)	18 (1.9%)	9.2 (2–19)	0.001	8.6 (2–19)	0.003
20–29 (ng/mL)	14 (1.5%)	7.1 (2–11)	0.003	7.5 (2–21)	0.010
≥ 30 (ng/mL)	2 (0.2%)	ref		ref	

Adjust model: Adjusted for age, pre-pregnancy BMI, parity, TCh, HDL, LDL and triglycerides

## Discussion

A few studies on vitamin D deficiency in Chinese pregnant women are after 20 weeks of pregnancy. Such as, the vitamin D deficiency rate (< 20 ng/ml) of pregnant women in Wuxi of China is 90% at 23–28 weeks of gestation [30], and the vitamin D deficiency rate (< 20 ng/ml) is 94.7% in Nanjing of China [31], and 90.2% in Beijing [21]. Our study population was a selected group of pregnant women who visited the outpatient clinic for their first prenatal examination when 12–14 weeks of gestation of Chinese pregnant women in Shanghai. Our study found over 97% of pregnant women had less than optimal levels of vitamin D level. Among all the 953 pregnant women, 691 (72.5%) were deficient, 239 (25.1%) were insufficient, and only 23 (2.4%) were sufficient. Our study results were consistent with other study results which the serum appropriate vitamin D level detection rate is less than 5% in pregnant Chinese women [12]. While vitamin D deficiency was high prevalence, the reason may be our serum samples were gathered during the winter and spring months, and part of the reason that beauty standards of Chinese culture are pale skin is sought-after amongst Chinese women, pregnant women put on more clothes to protect against ultraviolet rays during this period.

Our study found that the level of vitamin D of pregnant women was positively correlated with age and was negatively correlated with parity. Pregnant women under 30 and who had over 2 parturition experiences were more likely to suffer from vitamin D deficiency. Our study results showed that more attention should be given to this particular risky group of pregnant women, particularly under the Chinese government’s current Child Policy. In our study, overweight and obese pregnant women had lower vitamin D levels. There was a higher possibility of showing vitamin D insufficient based on the 30 nmol/l cutoff, indicating that they need to consume vitamin D supplements. Previous studies showed that daily vitamin D intake for overweight and obese adults should be 1.5 times and 2–3 times higher than those with normal weight [26, 32], indicating that it is essential for people with excess body weights to take vitamin D supplements.

Our study found that the increasing level of vitamin D nutritional status in pregnancy is significantly related to reducing the incidence of GDM. Vitamin D deficiency is a risk factor for GDM, consistent with some existing reports that maternal early pregnancy serum level of 25-Hydroxyvitamin D and risk of gestational diabetes mellitus [33–36]. The reason may be that vitamin D takes part in glucose homeostasis, and the mechanism possibly refers to insulin secretion and insulin action [37]. Moreover, a recent meta-analysis showed that maternal low vitamin D status accelerated the risk of GDM, lower vitamin D level increases the risk of type-2 diabetes and metabolic syndrome [38]. The reason also may be that vitamin D is known to impact insulin secretion. Vitamin D regulates insulin secretion by pancreatic β-cells and affects glucose levels in the circulation [39]. So, low vitamin D level is a risk factor for insulin resistance, glucose intolerance, and metabolic syndrome features in norm glycemic participants. Vitamin D deficiency during early pregnancy significantly increases the risk of gestational diabetes in late pregnancy [40]. Maintaining optimal vitamin D nutritional status during early pregnancy may be a protective factor for gestational diabetes mellitus.

Gestation is characterized by increased serum levels of total cholesterol and triglycerides pushed by estrogen, progesterone, and lactogen, which mobilize stored fat depots in late pregnancy, a pool of fatty acids for foetal growth, and placental tissue steroid synthesis. This physiologic increase in lipids performs an essential role during pregnancy; however, elevated lipids in susceptible women or familiar forms of hyperlipemia can increase maternal-foetal complications [41], and gestational hyperlipemia is associated with preeclampsia, preterm birth, and gestational diabetes, and offspring of these mothers show a propensity to enhanced fatty streak formation and an increased risk of progressive atherosclerosis [42]. It was previously demonstrated that chronically fructose-fed with diets containing high percentages of fructose develop hyperlipemia [43] and insulin resistance in pregnancy [44]. Vitamin D deficiency during pregnancy is associated with glucose and lipid metabolism, insulin resistance, and hyperlipemia [45]. Pre-eclampsia is higher in pregnant women with the vitamin D deficiency group than controls, and serum concentration of vitamin D is closely related to gestational diabetes and preterm delivery [46]. Our study found gestational hyperlipemia was a factor of vitamin D deficiency; however, there was no difference in vitamin D status between pregnancy of gestational hyperglycemia and normal pregnancy. This conflicting finding may have occurred because vitamin D and lipid metabolism in pregnancy is a complex process. Some studies reported that vitamin D supplements in pregnancy reduced serum total and LDL cholesterol levels but did not affect serum triglyceride and HDL-cholesterol levels [47, 48]. However, some researchers found vitamin D supplementation does not impact serum lipid profiles. In contrast, lipid profiles might decrease due to increased insulin sensitivity and parathyroid hormone reduction after vitamin D intake [49, 50]. Blood lipid impact on vitamin D levels was a complicated process, and more research was needed to explore the reason.

Our study has some limitations that should be mentioned. First, this study was a cross-sectional study; the associations it demonstrated do not imply a causal relationship between the level of vitamin D and risk in our study participants. Second, the study may be confounded by a lack of data on some relevant variables, such as dietary factors, especially vitamin D supplements, and different season factors. Moreover, this study did not perform a sample size calculation; therefore, the limited number of samples may affect our results’ statistical significance. Further studies are needed to evaluate the limitations of this study.

## Conclusions

In summary, there is a high prevalence of vitamin D deficiency in Chinese pregnancy in Shanghai. Aging more than 30 years, the parity of more than 2, overweight and obesity, and hyperlipemia are risk factors for vitamin D deficiency. Vitamin D deficiency is a risk factor for gestational diabetes mellitus. Public health strategies to prevent vitamin D deficiency should focus on those risks to promote healthy pregnancy in Shanghai in China.

## Data Availability

The datasets used and/or analysed during the current study are available from the corresponding author on reasonable request.

## References

[1] Millen AE, Bodnar LM (2008). Vitamin D assessment in population-based studies: a review of the issues. Am J Clin Nutr.

[2] Reid IR, Bolland MJ, Grey A (2014). Effects of vitamin D supplements on bone mineral density: a systematic review and meta-analysis. Lancet.

[3] Gorter EA, Krijnen P, Schipper IB (2016). Vitamin D deficiency in adult fracture patients: prevalence and risk factors. Eur J Trauma Emerg Surg.

[4] Borel P, Caillaud D, Cano NJ (2015). Vitamin D bioavailability: state of the art. Crit Rev Food Sci Nutr.

[5] Wang J (2018). Association between vitamin D deficiency and antepartum and postpartum depression: a systematic review and meta-analysis of longitudinal studies. Arch Gynecol Obstet.

[6] Bauer SR (2013). Plasma vitamin D levels, menopause, and risk of breast cancer: dose-response meta-analysis of prospective studies. Medicine (Baltimore).

[7] Cecchetti S (2016). Prevalence of vitamin D deficiency in rheumatoid arthritis and association with disease activity and cardiovascular risk factors: data from the COMEDRA study. Clin Exp Rheumatol.

[8] Azrielant S, Shoenfeld Y (2017). Vitamin D and the Immune System. Isr Med Assoc J.

[9] Walker VP, Modlin RL (2009). The vitamin D connection to pediatric infections and immune function. Pediatr Res.

[10] Biswas S (2018). Fok-I, Bsm-I, and Taq-I Variants of Vitamin D Receptor Polymorphism in the Development of Autism Spectrum Disorder: A Literature Review. Cureus.

[11] Brannon PM, Picciano MF (2011). Vitamin D in pregnancy and lactation in humans. Annu Rev Nutr.

[12] Yun C (2017). Vitamin D deficiency prevalence and risk factors among pregnant Chinese women. Public Health Nutr.

[13] Elsori DH, Hammoud MS (2018). Vitamin D deficiency in mothers, neonates and children. J Steroid Biochem Mol Biol.

[14] Yuan Y (2021). Association of maternal serum 25-hydroxyvitamin D concentrations with risk of preeclampsia: a nested case-control study and meta-analysis. J Matern Fetal Neonatal Med.

[15] Amraei M (2018). Effects of Vitamin D Deficiency on Incidence Risk of Gestational Diabetes Mellitus: A Systematic Review and Meta-analysis. Front Endocrinol (Lausanne).

[16] Bodnar LM (2010). Maternal serum 25-hydroxyvitamin D concentrations are associated with small-for-gestational age births in white women. J Nutr.

[17] Netting MJ, Middleton PF, Makrides M (2014). Does maternal diet during pregnancy and lactation affect outcomes in offspring? A systematic review of food-based approaches. Nutrition.

[18] Lee JM (2007). Vitamin D deficiency in a healthy group of mothers and newborn infants. Clin Pediatr (Phila).

[19] Bodnar LM (2007). High prevalence of vitamin D insufficiency in black and white pregnant women residing in the northern United States and their neonates. J Nutr.

[20] Yu X (2014). Vitamin D status and related factors in newborns in Shanghai. China Nutrients.

[21] Li H (2020). Prevalence of vitamin D deficiency in the pregnant women: an observational study in Shanghai. China Arch Public Health.

[22] Song SJ (2013). Vitamin D status in Chinese pregnant women and their newborns in Beijing and their relationships to birth size. Public Health Nutr.

[23] Kocak FE (2015). A comparison between two different automated total 25-hydroxyvitamin D immunoassay methods using liquid chromatography-tandem mass spectrometry. Biochem Med (Zagreb).

[24] Ross AC (2011). The 2011 report on dietary reference intakes for calcium and vitamin D from the Institute of Medicine: what clinicians need to know. J Clin Endocrinol Metab.

[25] Sempos CT, Binkley N (2020). 25-Hydroxyvitamin D assay standardisation and vitamin D guidelines paralysis. Public Health Nutr.

[26] Ekwaru JP (2014). The importance of body weight for the dose response relationship of oral vitamin D supplementation and serum 25-hydroxyvitamin D in healthy volunteers. PLoS ONE.

[27] Liguori A (2007). Effect of gestational hypercholesterolaemia on omental vasoreactivity, placental enzyme activity and transplacental passage of normal and oxidised fatty acids. BJOG.

[28] Leiva A (2011). Fetoplacental vascular endothelial dysfunction as an early phenomenon in the programming of human adult diseases in subjects born from gestational diabetes mellitus or obesity in pregnancy. Exp Diabetes Res.

[29] Giugliano D (2016). Comment on American Diabetes Association. Approaches to glycemic treatment. Sec. 7. In standards of medical care in diabetes-2016. Diabetes care 2016;39(Suppl. 1):S52-S59. Diab Care.

[30] Zhao X (2017). Maternal vitamin D status in the late second trimester and the rsk of severe preeclampsia in Southeastern China. Nutrients.

[31] Jiang L (2012). High prevalence of hypovitaminosis D among pregnant women in southeast China. Acta Paediatr.

[32] Bouillon R (2017). Optimal vitamin D supplementation strategies. Endocrine.

[33] Zhou Q (2020). Association between gene polymorphisms of vitamin D receptor and gestational diabetes mellitus: a systematic review and meta-analysis. Int J Environ Res Public Health.

[34] Iqbal S, Malik M, Bano G (2020). Serum Vitamin D levels and gestational diabetes mellitus: analysis of early pregnancy cohort from a teaching hospital of Kashmir Valley. J Family Med Prim Care.

[35] Yue CY, Ying CM (2020). Sufficience serum vitamin D before 20 weeks of pregnancy reduces the risk of gestational diabetes mellitus. Nutr Metab (Lond).

[36] Al-Shafei AI (2021). Maternal early pregnancy serum level of 25-Hydroxyvitamin D and risk of gestational diabetes mellitus. Int J Gynaecol Obstet.

[37] Wagner H (2016). No Effect of High-Dose Vitamin D Treatment on beta-Cell Function, Insulin Sensitivity, or Glucose Homeostasis in Subjects With Abnormal Glucose Tolerance: A Randomized Clinical Trial. Diabetes Care.

[38] Khan H (2013). Vitamin D, type 2 diabetes and other metabolic outcomes: a systematic review and meta-analysis of prospective studies. Proc Nutr Soc.

[39] Chiu KC (2004). Hypovitaminosis D is associated with insulin resistance and beta cell dysfunction. Am J Clin Nutr.

[40] Urrutia-Pereira M, Sole D (2015). Vitamin D deficiency in pregnancy and its impact on the fetus, the newborn and in childhood. Rev Paul Pediatr.

[41] Nasioudis D, Doulaveris G, Kanninen TT (2019). Dyslipidemia in pregnancy and maternal-fetal outcome. Minerva Ginecol.

[42] Cortés-Vásquez J, Noreña I, Mockus I (2018). Hypertriglyceridemia and adverse outcomes during pregnancy. Rev Fac Med.

[43] Rodriguez L (2013). Fructose during pregnancy affects maternal and fetal leptin signaling. J Nutr Biochem.

[44] Rodriguez L (2016). Fructose only in pregnancy provokes hyperinsulinemia, hypoadiponectinemia, and impaired insulin signaling in adult male, but not female, progeny. Eur J Nutr.

[45] Chen HY (2020). The relationship between maternal vitamin D deficiency and glycolipid metabolism and adverse pregnancy outcome. Clin Endocrinol (Oxf).

[46] Zhou J (2014). Associations between 25-hydroxyvitamin D levels and pregnancy outcomes: a prospective observational study in southern China. Eur J Clin Nutr.

[47] Longenecker CT (2012). Vitamin D supplementation and endothelial function in vitamin D deficient HIV-infected patients: a randomized placebo-controlled trial. Antivir Ther.

[48] Montero D (2014). Effect of antioxidant vitamin supplementation on endothelial function in type 2 diabetes mellitus: a systematic review and meta-analysis of randomized controlled trials. Obes Rev.

[49] Wang H (2012). Influence of vitamin D supplementation on plasma lipid profiles: a meta-analysis of randomized controlled trials. Lipids Health Dis.

[50] Jorde R (2010). No improvement in cardiovascular risk factors in overweight and obese subjects after supplementation with vitamin D3 for 1 year. J Intern Med.

